# Optimization of an Untargeted DART-HRMS Method Envisaging Identification of Potential Markers for Saffron Authenticity Assessment

**DOI:** 10.3390/foods10061238

**Published:** 2021-05-29

**Authors:** Elisabetta De Angelis, Rosa Pilolli, Alice Bejjani, Rocco Guagnano, Cristiano Garino, Marco Arlorio, Linda Monaci

**Affiliations:** 1 Institute of Science of Food Production, National Research Council of Italy, Via G. Amendola 126/O, 70126 Bari, Italy; elisabetta.deangelis@ispa.cnr.it (E.D.A.); rosa.pilolli@ispa.cnr.it (R.P.); rocco.guagnano@ispa.cnr.it (R.G.); 2 Lebanese Atomic Energy Commission, National Council for Scientific Research, Riad El Solh 107 2260 Beirut, Lebanon; a.bejjani@laec-cnrs.gov.lb; 3 Dipartimento di Scienze del Farmaco, Università degli Studi del Piemonte Orientale “Amedeo Avogadro”(UPO), Largo Donegani, 2, 28100 Novara, Italy; cristiano.garino@bfr.bund.de (C.G.); marco.arlorio@uniupo.it (M.A.); 4 German Federal Institute for Risk Assessment (BfR), D-14191 Berlin, Germany

**Keywords:** saffron, authenticity, adulteration, untargeted method, DART, mass spectrometry

## Abstract

Saffron is one of the most expensive agricultural products in the world and as such, the most commonly adulterated spice, with undeclared plant-based surrogates or synthetic components simulating color and morphology. Currently, saffron quality is certificated in the international trade market according to specific ISO guidelines, which test aroma, flavor, and color strength. However, it has been demonstrated that specific adulterants such as safflower, marigold, or turmeric up to 20% (*w/w*) cannot be detected under the prescribed approach; therefore, there is still a need for advanced and sensitive screening methods to cope with this open issue. The current investigation aims to develop a rapid and sensitive untargeted method based on an ambient mass spectrometry ionization source (DART) and an Orbitrap™high-resolution mass analyzer to discriminate pure and adulterated saffron samples with either safflower or turmeric. The metabolic profiles of pure and adulterated model samples prepared at different inclusion levels were acquired. Unsupervised multivariate analysis was carried out based on hierarchical cluster analysis and principal component analysis as first confirmation of the discriminating potential of the metabolic profile acquired under optimized DART-HRMS conditions. In addition, a preliminary selection of potential markers for saffron authenticity was accomplished, identifying compounds able to discriminate the type of adulteration down to a concentration level of 5%.

## 1. Introduction

Saffron is a precious and highly appreciated spice obtained in different Mediterranean regions by drying the stigmas of *Crocus sativus* L. (family *Iridaceae*). Due to its peculiar aromatic properties and color, it was used since ancient times in the food sector as a coloring and flavoring additive. In addition, several bioactive and pharmacologically active properties such as anti-diabetic [1], anticancer [2], immunomodulatory [3], analgesic [4], antimicrobial [5], antiatherogenic [6], cardioprotective [7], antioxidant [8], and anti-inflammatory [9] have been ascribed to saffron consumption. Iran, Greece, Morocco, Spain, and Italy are the largest saffron producers in the world, and beginning in 2015, the neighboring countries of Iran, such as Afghanistan, entered this market [10]. Among Mediterranean regions, Iran accounts for more than 90% of the saffron world’s production with Khorasan and Khorasan Razavi provinces being the main producers [10]. In Italy, saffron is cultivated mainly in Sardinia and Abruzzo and to a lesser extend in Umbria, Tuscany, Liguria, and Sicily. Environmental growing conditions and the local know-how adopted in the final thermal treatment particularly affects the final quality of *C. sativus* stigmas, also producing some peculiar characteristics that led some Italian productions (i.e., Abruzzese di Navelli and San Gimignano) to have been awarded the Protected Designation of Origin (PDO) quality mark.

Over 150 volatile compounds [11] together with non-volatiles molecules like flavonoids (such as glycosides of kaempferol and quercetin), carotenoids, isophorones, proteins, sugars, vitamins, carbohydrates were found to chemically characterize saffron [12,13]. Anyway, its peculiar properties were attributable to three main bioactive compounds, namely the water-soluble carotenoid crocin and its derivatives that are responsible for the golden yellow-orange color, picrocrocin responsible for the saffron bitterness [14], and finally safranal, a compound derived from the hydrolysis and oxidations of picrocrocin and responsible for the flavor originating during drying and preservation [13].

Due to the high costs and labor required for plantation and production, together with the huge number of flowers needed to obtain the spice, saffron could be considered one of the most expensive agricultural products of the world [15]. In the last years, its high value has prompted a rapid increase of fraudulent market so that saffron has been described as one of the most commonly adulterated food ingredients. It was found often fraudulently added with plant materials or synthetic components with similar color and morphology in order to increase its weight and/or improve its color properties [16]. Previous reports indicate that cut and/or dyed *Crocus sativus* stamens, *Calendula officinalis* L. petals (Calendula), *Carthamus tinctorius* L. petals (safflower), and *Curcuma longa* L. powdered rhizomes (turmeric) are among the most frequent adulterants in saffron [15,17,18,19].

Nowadays, saffron quality is certificated in the international trade market on the basis of its aroma, flavor, and color strength using the ISO 3632-1: 2011 method, which combines spectrophotometric measurements of picrocrocin and safranal, and chromatographic profiles of pigments (crocins) and apolar dyes that can be toxic (as Sudan dyes) [20,21]. Nevertheless, it has been demonstrated that saffron adulterants (safflower, marigold, or turmeric) up to 20% (*w/w*) were not detected by the ISO normative [22]. Therefore there is still a need for fast, simple, and robust screening methods suited for identifying saffron adulteration. Several analytical methodologies have been developed to detect plant adulterants in saffron samples spanning from molecular, spectroscopy, chromatographic, and biomimetic based techniques, as recently reviewed by Kiani et al., 2018 [23]. Although accurate and readily available, these techniques suffer from different drawbacks, such as loss of sample, time-consuming sample preparation, and normally they are not suitable for online quality analysis, especially in large scale and industrial applications [24,25]. In addition, most of them rely on a targeted approach where markers for adulterants were searched. A promising alternative strategy to detect saffron adulteration is offered by a metabolomics integrated approach which is characterized by an untargeted nature that allows comparative studies with samples classification by multivariate statistical analysis. Typical metabolic fingerprinting obtained by untargeted analysis or ambient mass ionization strategies should be also explored for discovering new emerging frauds or selecting molecules as markers for authenticity assessment. Non-targeted NMR-based metabolomics strategies have been employed to create models built on saffron metabolic profiles to assess its authenticity and/or detect adulterations [26]. Very recently, high-resolution mass spectrometry (HRMS) was successfully proposed for saffron authentication/traceability according to the geographical origin based on untargeted metabolic fingerprinting together with the identification of some peculiar markers [27]. Ambient mass spectrometry (AMS) is a relatively new field of analytical chemistry successfully applied to food authentication [28]. Since its introduction, almost 30 ambient ionization techniques for MS have been devised and presented [29,30]. Among them, desorption electrospray ionization (DESI), direct analysis in real-time (DART-HRMS), and atmospheric pressure solids analysis probe (ASAP) represents the techniques more established. Many papers investigated the feasibility of these techniques in food safety and authenticity fields, highlighting different performances for each of them [28]. Specifically, DESI has been demonstrated to be not particularly effective in detecting food adulteration, likely due to the lack of liquid chromatographic separation before MS detection [28]. As for ASAP, there are very few papers exploiting this technique to detect adulteration in foods, concluding that this approach provides good qualitative results, but concerning quantitative results, the technique struggles, thus being potentially insufficient for food fraud detection [28]. Conversely from what was observed for DESI and ASAP, a number of investigations demonstrated that DART-HRMS represents the most versatile approach to address food safety and authenticity issues [28,31,32], being fast, simple, and requires little or no sample preparation. Different mass analyzers have been hyphenated with DART source, with most of them acquiring satisfying results comparing with other conventional methods. High-resolution mass (HRM) analyzers such as time of flight (TOF), orbitrap, and linear ion trap (LIT) were often coupled with DART in order to obtain well-separated *m*/*z* molecular ion peaks with a consequent gain in limits of detection (LODs) and limits of quantitation (LOQs). Moreover, HRMass spectrometry represents one of the most suitable techniques for metabolic untargeted analysis and the identification of potential markers, thus representing a good tool for DART applications. Other mass analyzers such as quadrupole time-of-flight (QqTOF) and triple quadrupole (QqQ) can generate MS/MS fragment ion peaks useful for the detection and confirmation of unknown compounds [32].

In the past years, the DART-MS technique was successfully used for detecting food fraud in olive oil [33], beer [34], wine [35], animal fat [31], milk [36], and salmon [37]. As for spice, this approach was exploited for the authentication of true cinnamon [38] and for detecting the fraudulent presence of Japanese star anise in Chinese star anise and herbal teas commonly containing star anise [39].

To the best of our knowledge, such a technological platform was never applied to assess saffron authenticity. In the light of this, the current investigation aims to develop a rapid and sensitive untargeted method based on a DART source coupled with an Orbitrap mass analyzer to discriminate pure and adulterated saffron samples with *Carthamus* (safflower) and *Curcuma* (turmeric) inclusion down to concentration levels that would not be traceable with official ISO normative. In addition, the metabolic profile with and without the two adulterants was investigated in order to identify potential markers of the type of sophistication carried out.

## 2. Materials and Methods

### 2.1. Chemicals

Ethanol (≥99.8 GC) was purchased from Sigma Aldrich (Sigma Aldrich, Segrate, MI, Italy), while ultrapure water was produced by a Millipore Milli-Q system (Millipore, Bedford, MA, USA).

### 2.2. Sample Preparation

Pure Italian saffron powdered samples (*n* = 20) derived from a different region of origin (purchased and certified from Italian producers or Consortia of producers), along with saffron adulterated with safflower and turmeric at three different levels, namely 5%, 10%, and 20% (*n* = 3 each level), were analyzed for metabolic fingerprinting. Metabolite extraction was performed with the final aim to retain as many saffron metabolites as possible, thus obtaining the most comprehensive spectra applicable for discriminating between saffron pure and adulterated. The procedure described by Rubert et al., 2016 based on a solid-liquid extraction procedure was found to fit this purpose [27]. Briefly, 5 mL of ethanol/water (70/30, *v/v*) were added to 50 mg of powdered saffron followed by 1 h of shaker (KS 4000 i-control, IKA Works GmbH & Co. KG, Staufen, Germany) at 250 rpm, room temperature for increasing the extraction efficiency. After that, samples were centrifuged for 5 min at 13,416× *g* (20 °C), and the supernatant was collected for DART-HRMS analysis.

### 2.3. DART-HRMS Analysis

DART–HRMS analyses were performed using a DART–HRMS system consisting of a DART SI-140-GIST (DART Thermo Ion Max Vapur Interface, Ion Sense Inc., Saugus, MA, USA) coupled to Exactive^TM^ non-hybrid single-stage Orbitrap mass spectrometer (Thermo Fisher Scientific, San Jose, CA, USA). The ion source and the mass spectrometer were hyphenated by a Vapor gas ion separator interface (Ion Sense Inc., Saugus, MA, USA), while a low vacuum in the interface chamber was maintained by a membrane pump (VACUUBRAND GMBH + CO KG, Wertheim, Germany). The DART–HRMS instrument was operated in positive or negative ionization mode according to the settings detailed in Table 1.

Sheath, auxiliary, and sweep gases were disabled during the analysis. The mass spectra were recorded in the *m*/*z* range 100–1000, and the maximum injection time was set to 250 ms. The mass resolving power of the instrument calculated at *m*/*z* 200 was 50,000 FWHM (full width at half maximum), and Microscan was set to 2. The ethanol-aqueous extracts of pure and adulterated saffron samples were delivered to the DART ionization region with the use of a 12 Dip-It tip scanner autosampler. Twelve Dip-It tips (IonSense, Saugus, MA, USA) were inserted into a holder and immersed in sample extracts placed in a 500 µL centrifuge tube. After mounting the holder on the body of the autosampler, the Dip-It tips were set to automatically move at a constant speed of 0.5 mm/s through the helium gas stream delivered from the DART gun exit in order to promote sample desorption. The time of desorption from the surface of each tip was 6 s, and three parallel analyses (three samples independently extracted for each saffron powder for both pure and adulterated type) were performed for each saffron pure and adulterated sample. Along with DART mass spectra, peculiar chronograms, namely a representation of ions desorption during the time of mass spectra acquisition, were obtained for each sample analyzed. The figure is produced by the software, and its curve-shape derived from the ions desorption trend that increases over time up to a maximum value and then decreases down to zero.

### 2.4. Data Processing and Statistical Analysis

The MS fingerprints from DART-HRMS analysis of saffron pure and adulterated with safflower and turmeric were acquired in positive and negative mode using Xcalibur software (version 2.1, Thermo Fisher Scientific, San Jose, CA, USA), while MS data processing for small compound identification and chemometric analysis, such as peaks alignment and features extraction, were performed using Compound Discoverer software (version 3.1.1.12, Thermo Fisher Scientific, San Jose, CA, USA). The values of the relerelevant parameters for features extractions were the following: precursor ion deviation of 5 ppm (both for positive and negative runs); maximum retention time shift of 2 min; minimum peak intensity for a peak to be taking into account 10,000 AU; relative intensity tolerance used for isotope search 30% and S/N larger than 3.

In order to filter the compound list to the species most suitable in discriminating pure saffron from those adulterated, Volcano plots combining the statistical significance of the identified compounds and magnitude of change in the extracted peak areas were investigated. Compounds lists were constrained according to *p*-value thresholds ≤0.05 and ≤0.01 applied to every ratio analyzed, namely pure saffron/safflower adulteration, pure saffron/turmeric adulteration, and safflower/turmeric adulterants. The edited compounds list was finally processed by hierarchical cluster analysis-heat map (HCA) in order to group samples according to their intrinsic similarities and unsupervised multivariate modeling, namely Principal Component Analysis (PCA), to inspect any natural grouping between pure and adulterated saffron. As for HCA, the following parameters were set: distance function = Euclidean, linkage method = Ward, scale values: Before clustering.

Finally, potential discriminating compounds were selected from the Volcano plot and PCA loading plot and putatively identified by the Chemspider platform as well as the entire isotopic profile of the target compounds obtained by activating the workflow node “apply spectral distance” (mass accuracy 5 ppm for each centroid mass in the isotopic cluster). Chemspider databases including ChEBI, ChemBank, ChEMBL, ChemMine, DrugBank, FDA, FooDB, LipidMaps, carotenoids database, Nature Chemistry, Peptides, Phenol-Explorer, PubMed, setting a maximum mass shift of 5 ppm for positive and negative acquisitions were queried for compounds identification. Molecule identification was carried out according to the criteria set out by the Metabolomics Standard Initiative [40,41]. Specifically, the standard criteria ‘level III’ (putatively characterized compounds), corresponding to compounds identified by HR-MS and by spectral similarities from databases and literature, was fulfilled.

## 3. Results

### 3.1. DART-HRMS Conditions Optimization

In the first phase of experiments, DART settings were optimized in order to collect wide information on the chemical composition of the samples. To this aim, DART ionization was tuned towards the highest number of detected ions by exploring the acquisition range of 100–1000 *m*/*z*. This last was selected on the basis of DART mechanisms of ionization which results effectively mostly for small molecules with molecular weights (MW) not exceeding 1 kDa [42]. The method was optimized by analyzing pure saffron extracted by a mixture of ethanol/water, as detailed in the Materials and Methods section. The first tuned parameter during the optimization of the DART detection process was the ionization temperature that previous works demonstrated to have a dramatic impact on the DART mass spectra quality [43,44]. In positive mode, three temperature values were investigated, namely 250, 350, and 450 °C. Results obtained revealed that the number of detected ions were found to increase by approximately 2.5 times as the temperature raised up to 450 °C. Therefore this value was chosen as the optimal setting for the next experiments. On the contrary, 350°C was found as an optimal compromise between signal intensity and thermal desorption speed of analytes for DART analysis in negative mode. The most important MS parameters (capillary temperature, capillary voltage, tube lens voltage, and skimmer voltage) were optimized as well both in positive and negative ion mode by carrying out dedicated experiments on pure saffron extract. As for positive ion acquisition, the capillary temperature was explored in the range of 200 and 250 °C. This last condition enabled a 55% increase in the number of signals when compared to low temperature, therefore it was chosen for the next analysis. The capillary voltage was tested in the range of 20 and 55 V, and, in this case, the highest number of ions detected was obtained by setting the voltage to 55 V. The impact of tube lens voltage on the final signal of produced mass spectra was investigated in the range of 65 and 130 V and this last condition was found to be the most appropriate for saffron analysis. As for skimmer voltage, the value of 26 V was identified as the best parameter. Regarding mass spectra acquisition in negative mode, the impact of each MS parameter, mentioned above, was investigated in the same range explored for positive acquisition. The final setting was nearly different, as detailed in Table 1.

**Figure 1:**
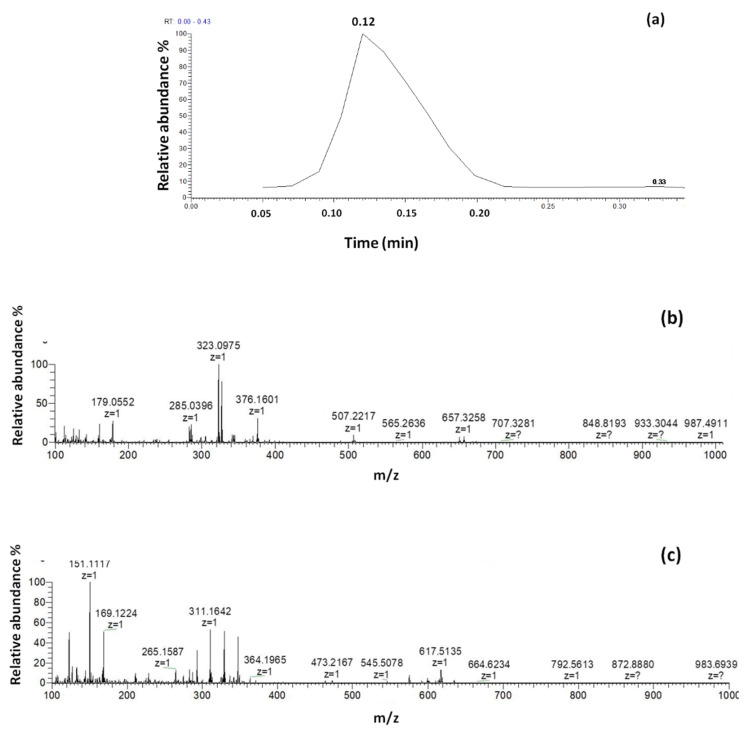
Chronogram (**a**) along with mass spectra obtained by DART-HRMS analysis of pure saffron in negative (**b**) and positive (**c**) ion mode acquisition with the optimized conditions.

The mass spectra of extract prepared from pure saffron recorded in positive and negative mode, along with the chronogram obtained by the optimized method, are shown in Figure 1.

### 3.2. Data Processing and Chemometric Analysis

As previously mentioned, pure saffron samples and saffron spiked with different amounts of safflower and turmeric adulterants (5-10-20% level for each adulterant) were extracted with a mix of ethanol and water and analyzed by the DART-HRMS optimized method in both positive and negative modes. The final MS fingerprints obtained were investigated to detect food adulteration by statistical analysis. Firstly, the produced spectra were processed via the commercial software Compound Discoverer v.3.1.1.12 SP1 (Thermo Fisher Scientific). In particular, the chronograms were aligned, and relevant ions were grouped and identified with a mass accuracy of ≤5 ppm. The type of adulteration carried out (safflower and turmeric) was set as the categorical factor for data analysis and sample discrimination by unsupervised multivariate statistical analysis. As the first level of investigation, the DART fingerprints obtained by the three levels of adulteration (5, 10 and 20%) were grouped together as part of the same class (adulterated samples) for either turmeric or safflower addition. Thus, three main data groups were obtained and compared, namely pure saffron, saffron adulterated with turmeric, and saffron adulterated with safflower.

An ANOVA statistical test was performed in order to highlight main compounds potentially able to discriminate pure saffron from adulterated samples. Volcano plots combining the magnitude of change in the extracted peak areas and their statistical significance were investigated. The list of compounds with significant differences among groups were constrained by *p*-value threshold (≤0.05) filtration and for DART-MS spectra acquired in positive mode, a total of 50 features were selected. It is worthy to be noted that by activating the option “every ratio”, only the features fulfilling the *p*-value restriction simultaneously in the three-ratio investigated (pure saffron/safflower, pure saffron/turmeric, and safflower/turmeric adulterants) were taken into account.

**Figure 2:**
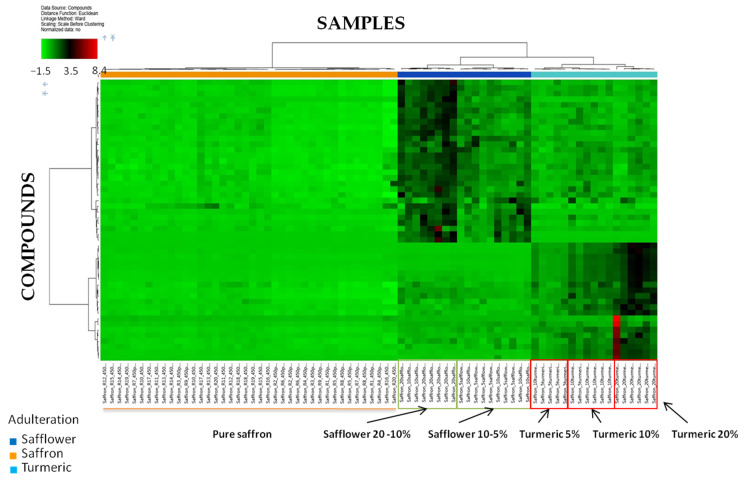
Hierarchical Cluster Analysis of the peak areas recorded for the compounds list obtained after filtration of the software output by *p*-value ≤0.05 in every ratio of the ANOVA test.

Filtered data were processed via Hierarchical Cluster Analysis (HCA) in order to visualize the correlation between detected compounds and selected samples in a two-dimensional array of color-coded rectangles (heat map) where each rectangle represents the relative amounts (by area) of a specific compound in a specific sample. The application uses an agglomerative (bottom-up) approach to find the similarities between samples and compounds. Initially, the hierarchical cluster analysis assigns each compound to its own singleton cluster. The analysis then proceeds iteratively, at every single stage joining the two most similar clusters into a new cluster, continuing until there is only an overall cluster represented by a dendrogram. The results are shown in Figure 2.

The heat map of Figure 2 shows the capability of the metabolic profiles acquired to discriminate not only pure saffron samples from adulterated ones but also the ability to discriminate different types of adulteration (either with safflower or turmeric), proving to be a potentially useful tool for food traceability. Indeed, the data set appeared clustered in three main groups according to the color codes: (i) pure saffron samples (marked with orange bar), (ii) saffron samples including variable amounts of safflower (5, 10, and 20%, marked with blue bar), (iii) saffron samples adulterated with turmeric (5, 10, and 20%, marked with cyan bar). In fact, the heat map from HCA (for reference, see the upper part of Figure 2, on the left, where is reported a color-scale indicating the fold of change of ions peak areas spanning from approximately −1.5 and 8.4, as calculated by the software, each ion is colored in a different way according to its area value) showed that compared to pure saffron, counterfeit samples are characterized by specific compounds whose area increases up to approximately 3.5 fold by passing from 0 to 5, 10, and 20% inclusion levels with some compounds of turmeric even showing an area 8.4 higher with respect the correspondent pure saffron. In addition, a good separation is visible between samples mixed with the two different adulterants, highlighting that the features selected are the most appropriate to discriminate simultaneously between pure saffron and the two adulterant species. As for the identification of the levels of inclusion in adulterated samples, different compounds allowing a good separation between pure saffron and each investigated level were observed for turmeric, as demonstrated by the red boxes of Figure 2, which show the natural grouping of the samples differently adulterated at the three different levels. On the contrary, although it seems possible to discriminate saffron pure from that adulterated with safflower even at a low level, no clear classification was observed between 5 and 10% inclusion levels that, instead, appears well separated from the 20% adulteration (see the green boxes on Figure 2).

These results are confirmed by the HCA dendrograms reported in Figure S1A,B of Supplementary Material, where the distance calculation between clusters was shown. As appears by the HCA dendrogram reported in Figure 1a, pure saffron samples clustered in different little groups according to their similarities with the maximum distance between the two final clusters calculated as 12.77, which suggests a good similarity between the overall samples. As for saffron adulterated with safflower and turmeric, two distinct classes are visible in Figure 1b (marked with blue bar-safflower and cyan bar-turmeric) well separated each other and from pure saffron. In particular, saffron samples enriched with safflower or turmeric resulted clustered in two main groups among which the maximum distance calculated was 92.51, highlighting that the fraudulent addition of safflower or turmeric differently changes the final metabolic profile of saffron. At a general level, the distance between pure saffron and adulterated ones was likely higher than 92.51, thus suggesting a good capability of the model to discriminate pure samples from that counterfeit. It is worthy to note that for turmeric adulteration, three different clusters, each one referred to the three different levels of inclusion, are noticeable in the respective dendrogram (Figure S1B Supplementary Material), although a more marked distance was obtained for 20% level. These results confirm what was already observed by analyzing heat map analysis.

**Figure 3:**
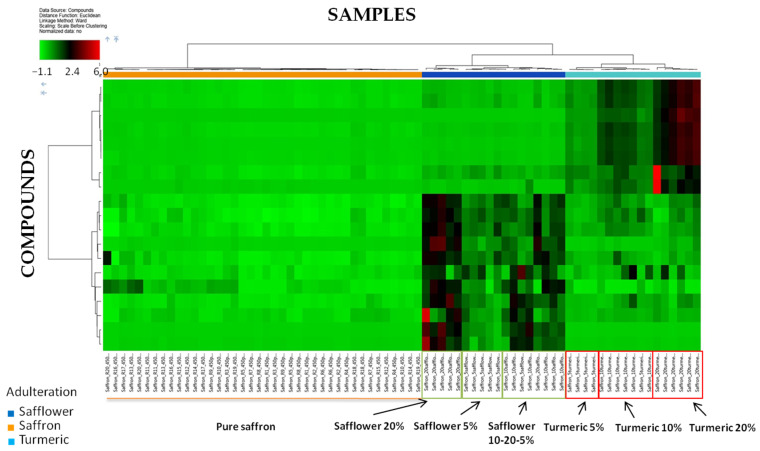
Hierarchical Cluster Analysis of the peak areas recorded for the compounds list obtained after filtration of the software output by *p*-value ≤0.01 in every ratio of the ANOVA test.

In order to identify the compounds that contribute mostly to the discrimination of pure saffron from those adulterated, the list of features resulting from ANOVA analysis was further refined by selecting only the input variables fulfilling the criteria *p*-value ≤ 0.01 in every ratio. Only 19 features were selected by the software and processed via HCA analysis (Figure 3).

Results are similar to that already discussed for HCA obtained with a higher *p*-value threshold, namely a clear separation within pure and adulterated saffron. Turmeric and safflower enriched samples are separated from each other, and a very good classification between low, middle, and high inclusion levels was obtained for turmeric samples, for which several compounds, whose area increases up to six-fold by passing from 0 and 5 to 20% adulteration could be noted. As for safflower samples, the classification between the three inclusion levels barely improved, although a clear separation for the 10% level is still missing. For turmeric adulterated samples, different species whose area increased up to six-fold, from pure saffron up to 20% levels were observed, highlighting their likely contribution in the discrimination of the classes. The HCA dendrograms with the distance calculation between clusters for pure saffron and adulterated samples are separately shown in Figure S2 of Supplementary Material. As for pure saffron (Figure S2, panel A Supplementary Material) the maximum distance calculated between the clusters obtained by the progressive grouping of analyzed samples was 8.30, suggesting once again a good similarity among the samples. As for the counterfeit samples, the maximum distance calculated between the two different classes of adulterants was 66.43, as shown in the dendrogram illustrated in Figure S2, panel B of Supplementary Material. A distance higher than 66.43 is supposed to separate clusters of pure saffron and adulterated samples, thus confirming the results previously discussed.

Similar data analysis was carried out also on MS/MS spectra acquired in negative analysis mode. However, no significant discrimination was observed by HCA (data not shown), thus this data set was excluded by further discussion.

**Figure 4:**
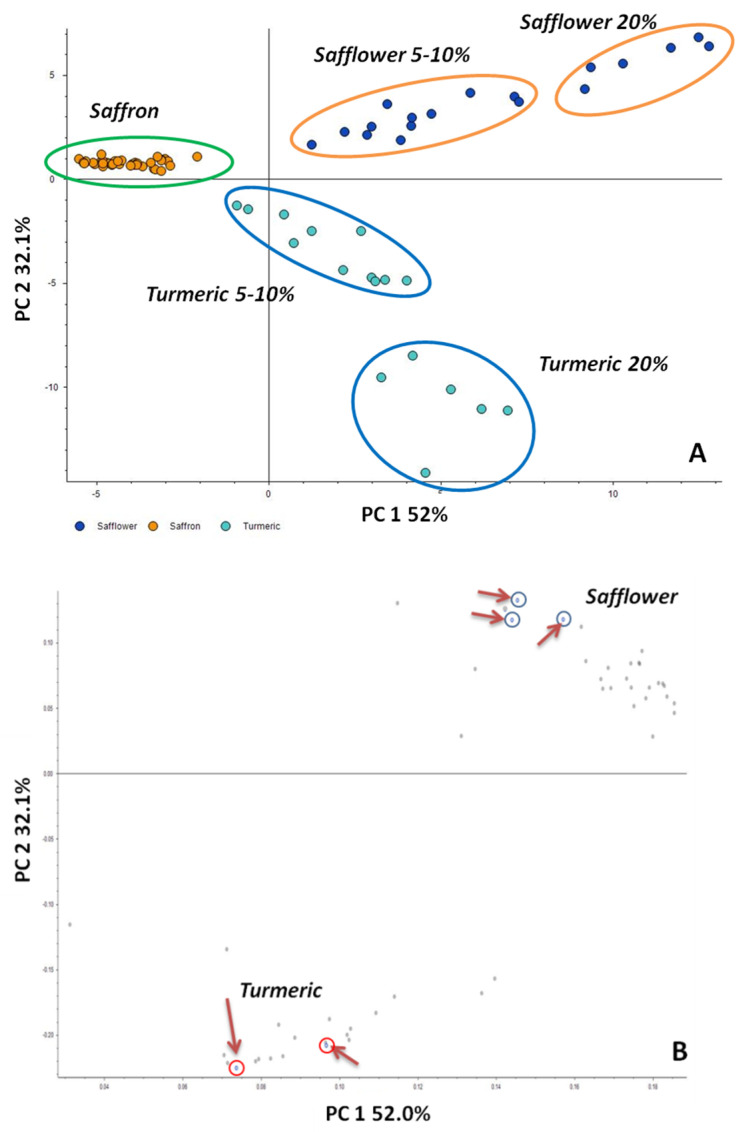
Principal component Analysis (PCA) scores plot (**A**) and loading plot (**B**) obtained for positive ionization mode after data filtration by a *p*-value threshold (≤0.05). Data points which strongly contribute to both principal components are target with arrows in the loading plot.

As the next step, metabolomics data acquired by DART-HRMS was subjected to principal component analysis (PCA). PCA represents a widely used unsupervised pattern recognition technique, which allows visualization of the multidimensional information in the form of a few principal components retaining the maximum possible variability within the data set. Similarly to what was described for the HCA test, the PCA model was built up by using only the input variables fulfilling the criteria of *p*-value ≤ 0.05 as resulting by the ANOVA test performed by comparing pure and adulterate saffron samples (either by safflower or by turmeric) analyzed in positive ion mode. PCA results were reported in Figure 4 as a score plot (panel A) which depicts the location of samples in a bi-dimensional space, and the loading plot, which illustrated the compound distributions (panel B).

As shown in Figure 4, a good clustering was obtained between the three groups explored, indeed PC1 and PC2 together describe 84.1% of the sample set variability (52% and 32.1% for the PC1 and PC2, respectively). Considering the fact that the first three PCs explain 89.4% of the total variance, the PC1/PC2 plot seemed to be a good starting point for sample clustering according to their purity. The proper distinction between samples of saffron pure and adulterated was allowed even considering the type of adulterant. Indeed, saffron samples mixed with turmeric and safflower are grouped in different clusters, and for both of them, the 20% inclusion level is clearly discriminated from 5 and 10% adulteration percentages that, on the contrary, resulted not clustered. These results highlight that the metabolic profile designed was able to discriminate between pure and adulterated saffron samples with either turmeric or safflower even at the low level of inclusion (5%) although, there is not a clear separation between the three levels of adulteration. As it can be seen in Figure 4, pure and adulterated groups were clearly separated, and the most significant compounds, which are responsible for the separation, can be also seen in the loading plot (see the blue dots marked with red arrows and small circles, Figure 4b). The loading plot represents the importance of markers, and molecular features close to the intersection are less significant than remote ones. According to PCA Loading Plots, three main compounds were responsible for grouping between saffron and safflower, having *m*/*z* 409.3817, *m*/*z* 409.4030, and *m*/*z* 303.2311, as resumed in Table 2.

**Figure 5:**
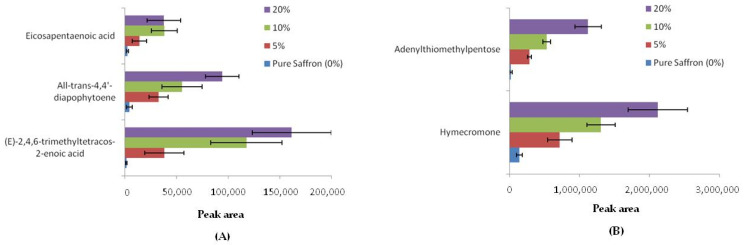
Signal intensity variation of candidate marker compounds among the inclusion levels of adulteration explored: 0 (pure saffron), 5, 10 and 20%: (**A**) safflower markers, (**B**) turmeric markers.

The first signal was detected as [M+H]^+^ and tentatively annotated as all-trans 4,4′diapophytoene corresponding to the chemical formula C_30_H_48_ and belonging to the apo carotenoid triterpenoid family, while *m*/*z* 409.4030 corresponds to the protonated form of E-2,4,6-trimethyltetracos-2-enoic-acid ([M+H]^+^, chemical formula C_27_H_52_O_2_), a branched-chain fatty acid with methyl branching at C-2, -4 and -6, and with a double bond at C-2. The third signal with *m*/*z* of 303.2311 was detected as [M+H]^+^ and putatively attributed to eicosapentaenoic acid (chemical formula of C_20_H_30_O_2_), an important polyunsaturated fatty acid classified among the omega-3 fatty acids. As for turmeric, only two compounds were identified as putative markers (see Table 2), namely hymecromone (*m*/*z* 177.0642, chemical formula C_10_H_8_O_3_) detected in the protonated form [M+H]^+^ and adenylthiomethylpentose with *m*/*z* 339.1218 (chemical formula C_11_H_15_N_5_O_3_S) detected in positive mode as an adduct with acetonitrile [M+AcN+H]^+^. The first molecule is a hydroxycoumarin, namely umbelliferone substituted by a methyl group at position four, while the adenylthiomethylpentose is a sulfur-containing nucleoside. In all the *m*/*z* assignment, error mass ppm was lower than three, and only the compounds with spectral similarity score between the measured and theoretical isotope pattern higher than 75% were taken into account, with the only exception of adenylthiomethylpentose for which a mass accuracy of 4.31 and a SFit% of 60% were calculated. In Table 2, the variation of the signal intensity observed for each candidate marker by passing from pure saffron to adulterated saffron with safflower or turmeric were depicted as a heat map. The color of each box depends on the area recorded for the corresponding molecule and ranges between red and green, where red refers to the lower value. As appears from the table, the signal of each selected compound increases as the inclusion level of adulteration rise, meaning that all putative markers are likely compounds typical of safflower or turmeric metabolome. The signal intensity of each compound was found to markedly increase by comparing saffron pure and adulterated at 5% inclusion level, both in the case of safflower or turmeric counterfeit, suggesting that these species could allow to reveal adulterated saffron even at the lowest level tested. In Figure 5, the contribution of each compound to discriminate between pure and adulterants included at different levels was depicted in detail.

As for safflower (Figure 5A), it clearly appears that all the three compounds allowed to discriminate between saffron pure and adulterated at a 5% level; indeed, an increase of the respective peak areas ranging between 82 and 96% was observed by comparing the two samples. On the contrary, the same compounds were found less reliable in the discrimination among the different inclusion levels of safflower. Only the peak area of (E)-2,4,6-trimethyltetracos-2-enoic acid at 5% levels seems statistically different from the respective 10% level, but no candidate compounds appear to effectively distinguish between 10 and 20% inclusion level. On the contrary, concerning turmeric adulteration, more promising results could be observable in Figure 5B. Both hymecromone and adenylthiomethylpentose molecules were found to increase their peak intensity by 80 and 90%, respectively, in a 5% inclusion level with respect to the pure counterpart. In addition, their peak area was observed to rise by approximately 50% by passing from 5 to 10 and from 10 to 20% levels, highlighting that the targeted analysis of these molecules could be useful to investigate within the same run the levels of adulteration of saffron with turmeric. 

The issue of saffron adulteration was extensively studied, and different analytical methods relying on targeted and untargeted approaches were proposed in the literature [23]. Many analytical techniques were explored for discriminating between saffron pure and counterfeit, such as chromatography, spectroscopy, molecular-biological techniques, and biomimetic-based techniques [23]. Very recently, mass spectrometry coupled with chemometric tools was successfully investigated to develop reliable methods based on metabolomics approach (comprehensive non-targeted screening of metabolites, especially small molecules up to 1200 Da in molecular weight), able to discriminate saffron pure from adulterated. Rubert et al. in 2016 developed an analytical method based on an untargeted metabolic fingerprinting approach employing UHPLC–HRMS merged with chemometrics to distinguish saffron origin [27]. The supervised and unsupervised models proposed distinguished between (Protected Designation of Origin) PDO saffron and labeled Spanish saffron, demonstrating fraudulent behavior in more than 50% of samples. Moreover, by exploiting the powerful tool of high resolution by mass spectrometry, different compounds belonging to glycerophospholipids and their lipid oxidation products were identified and tentatively confirmed as the most significant markers to discriminate PDO Spanish saffron [27]. In addition, in 2019, Senizza et al. proposed a UHPLC-ESI/QTOF-MS method based on the metabolomic approach followed by statistical multivariate analysis to investigate the discrimination potential between adulterated (added with different percentage of other parts of the flower) and authentic saffron, as well as to trace its geographical origin [45]. In particular, the phenolic fraction was taken into account. Both unsupervised (hierarchical clustering) and supervised orthogonal partial least squares discriminant analysis allowed discriminating authentic saffron from styles added of other floral components starting from an inclusion level of 5%. PDO vs. non-PDO saffron samples were separated according to their chemical fingerprints as well. Moreover, different validated markers belonging to anthocyanins and glycosidicflavonols were proposed for the styles’ adulteration. While for the discrimination of PDO vs. non-PDO saffron samples, other flavonoids compounds (mainly free flavonols and flavones), together with protocatechuic aldehyde and isomeric forms of hydroxybenzoic acid, were validated as markers [45]. In the present study, we suggest the potential use of DART–HRMS in the determination of saffron adulteration with safflower or turmeric, reducing the time required for the analysis compared to chromatographic techniques. Both the unsupervised (hierarchical clustering and PCA analysis) multivariate statistics explored allowed discriminating authentic saffron from the counterfeit counterparts starting from the 5% level of inclusion. Moreover, after statistical treatment, different marker compounds were putatively identified exploiting the high-resolution tool offered by the MS equipment used coupled with advanced statistical software.

## 4. Conclusions

A reliable method for saffron authentication and integrity was successfully developed to discriminate pure saffron from adulterated saffron with safflower or turmeric. The method was based on DART ionization coupled with an untargeted HRMS approach followed by chemometrics analysis. Pure saffron and saffron adulterated with safflower (*C. tinctorius*) or turmeric (*C. longa*) were effectively discriminated, as demonstrated by HCA and PCA analysis performed on data filtered on ANOVA *p*-value threshold, for MS spectra acquired in positive ion mode. In particular, the adulteration of saffron with safflower or turmeric was well characterized starting from low inclusions level, namely 5–10%. Generally speaking, a clear separation was obtained between the two macro groups composed of pure and adulterated samples, while no clear distinction was always observed within the classes each referred to the different adulterant inclusion levels investigated, both for safflower and turmeric species. If effective discrimination was observed for 20% inclusion level compared with other classes, samples adulterated at 5–10% levels are grouped together, suggesting that DART metabolic profiles are not still enough different to produce a significant separation between samples adulterated at these percentages. In light of this, a future effort will be directed to develop a predictive model able to functionally discriminate authentic saffron from adulterated one at different inclusion levels. Moreover, potential markers to search fraudulent addiction of safflower and turmeric in saffron were putatively identified with some of them also allowing the identification of the adulteration level. Our results demonstrated the reliability of the DART-HRMS unconventional fingerprinting-based approach in a future industrial scenario to evaluate the risk of substitution of higher value saffron with lower value species, such as safflower and turmeric.

## Figures and Tables

**Table 1:** DART parameters and HRMS conditions for untargeted analysis of pure and adulterated (with safflower and turmeric) saffron samples.

Analysis Mode (Polarity)	DART Parameters		MS Conditions			
	Gas T °C	Grid V	Capillary T °C	Capillary V	Tube Lens	Skimmer V
Positive	450	250	250	55	130	26
Negative	350	−350	200	−50	−120	−25

**Table 2:** Candidate marker compounds to discriminate between saffron pure and adulterated with safflower or turmeric at different inclusion levels (0, 5, 10 and 20%).

Adulterant	MS Characterization						Level of Inclusion (Signal Intensity)			
	Compound	Chemical Formula	Mass Accuracy (ppm)	*m*/*z*	Adduct	SFit%	0%	5%	10%	20%
Safflower	€-2,4,6-trimethyltetracos-2-enoic acid	C_27_H_52_O_2_	−2.56	409.4030	[M+H]^+^	84				
	All-trans-4,4′-diapophytoene	C_30_H_48_	−2.28	409.3817	[M+H]^+^	75				
	Eicosapentenoic acid	C_20_H_30_O_2_	−2.12	303.2311	[M+H]^+^	95				
Turmeric	Hymecromone	C_10_H_8_O_3_	−1.41	177.0642	[M+H]^+^	85				
	Adenylthiomethylpentose	C_11_H_15_N_5_O_3_S	−4.31	339.1218	[M+AcN+H]^+^	60				
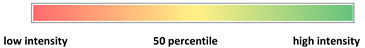										

## Supplementary Materials

The following are available online at https://www.mdpi.com/article/10.3390/foods10061238/s1. Figure S1: Hierarchical Cluster Analysis (HCA) of compounds list of pure saffron (A) and adulterated samples (B) obtained after filtration of the software output by *p*-value ≤ 0.05 in every ratio of the ANOVA test. In particular, the dendograms clusterizing the different samples along with the distances calculated for the main nodes were reported; Figure S2: Hierarchical Cluster Analysis (HCA) of compounds list of pure saffron (A) and adulterated samples (B) obtained after filtration of the software output by *p*-value ≤ 0.01 in every ratio of the ANOVA test. In particular, the dendograms clusterizing the different samples along with the distances calculated for the main nodes were reported.
