# Supplementary material for: Optimization of an Untargeted DART-HRMS Method Envisaging Identification of Potential Markers for Saffron Authenticity Assessment
Source: Foods. 2021 May 29;10(6):1238. doi: 10.3390/foods10061238 (PMC8230169; doi:10.3390/foods10061238)
Supplement: Supplementary file 1 [file foods-10-01238-s001.zip › foods-1209219-supplementary.pdf]

Figure 1 Supplementary. Hierarchical Cluster Analysis (HCA) of compounds list of pure saffron (A) and adulterated samples (B) obtained after filtration of the software output by p-value ≤0.05 in every ratio of the ANOVA test. In particular, the dendograms clusterizing the different samples along with the distances calculated for the main nodes were reported.

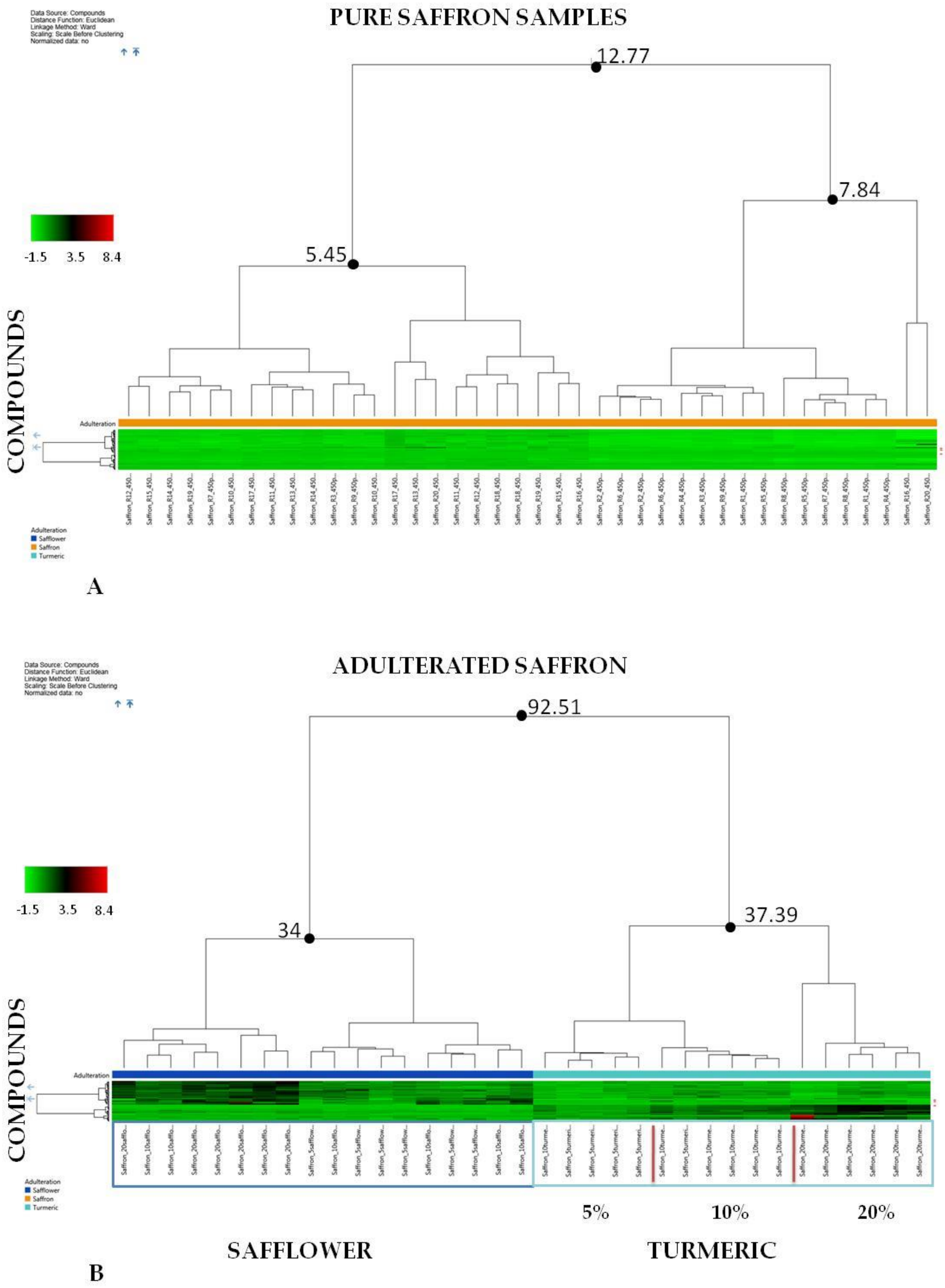

Figure 2 Supplementary. Hierarchical Cluster Analysis (HCA) of compounds list of pure saffron (A) and adulterated samples (B) obtained after filtration of the software output by p-value ≤0.01 in every ratio of the ANOVA test. In particular, the dendograms clusterizing the different samples along with the distances calculated for the main nodes were reported.

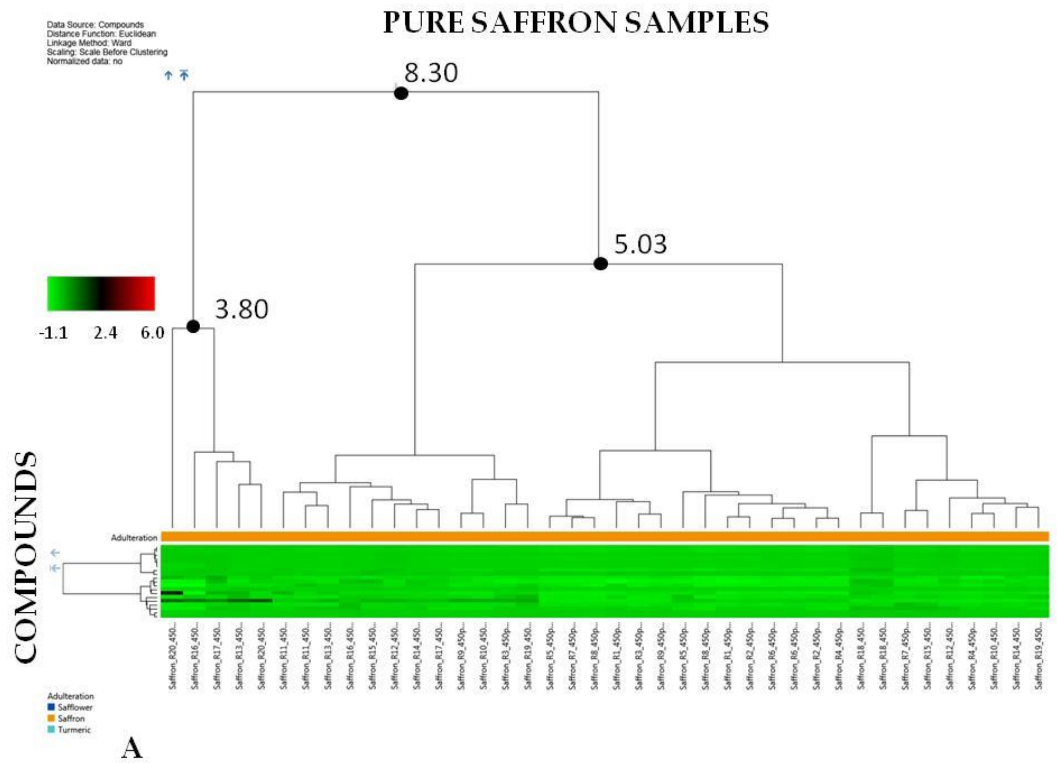

A

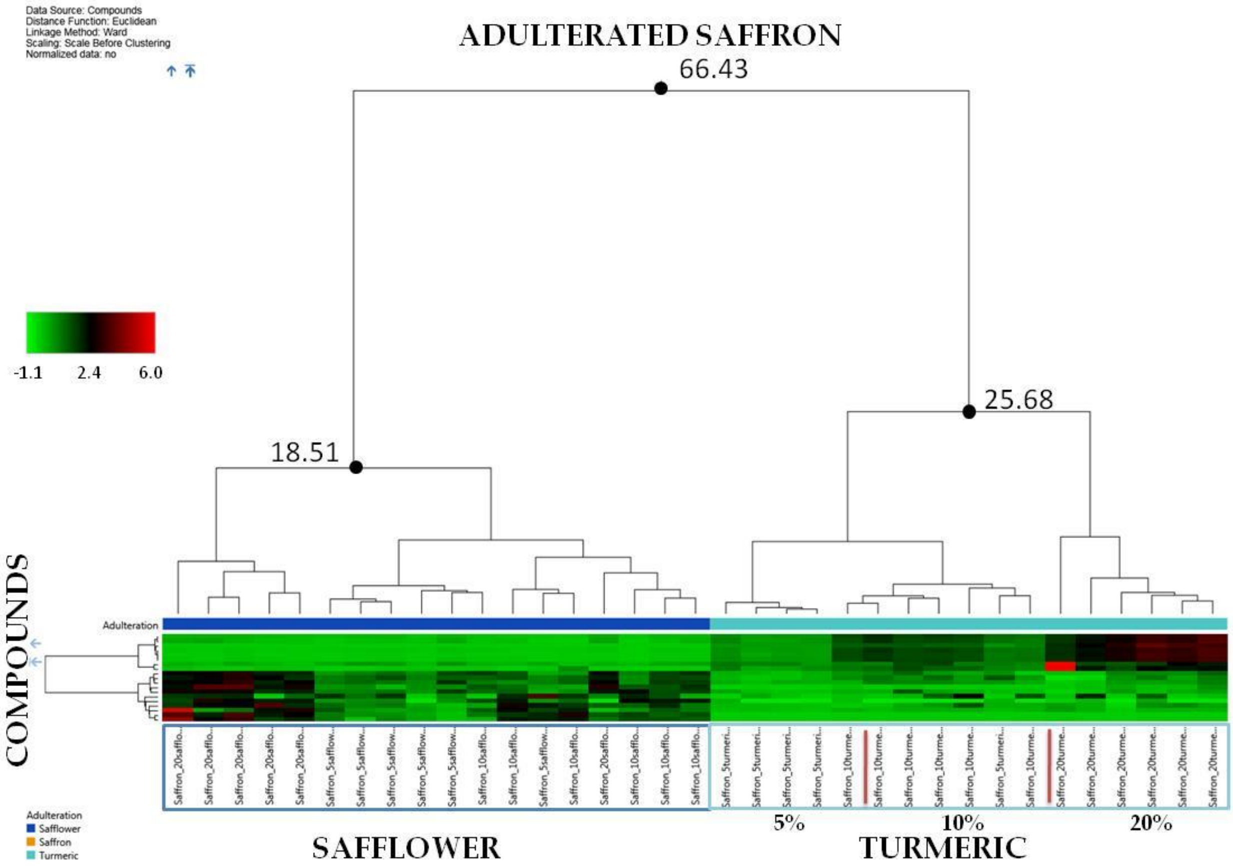
Data Source: Compounds
Distance Function: Euclidean
Linkage Method: Ward
Scaling: Scale Before Clustering
Normalized data: no
ADULTERATED SAFFRON
66.43
18.51
25.68
-1.1
2.4
6.0
COMPOUNDS
Adulteration
5%
10%
20%
SAFFLOWER
TURMERIC
Adulteration
Safflower
Saffron
Turmeric

B
